# Carcinoma

**Published:** 1888-12

**Authors:** John V. Allen

**Affiliations:** Philadelphia


### CARCINOMA.

John V. Allen, M. D., Philadelphia.

As it is my fortune to have at this time a case of carcinoma of the throat which seems to tally in its details with those of the Emperor of Germany, I thought it well to bring before the Society's notice the treatment (homœopathic) which my patient receives, and the progress toward a cure which the same is developing. The condition of the Emperor is bulletined daily throughout the world, as is also the heroic treatment which he receives from the greatest specialists of Europe, while the condition of my patient is known only by his townsmen, and has only a Hahnemannian, and not a particular specialist, to handle his case.

But the improvement is noticeable and continual under the latter's treatment, while we hourly look for the demise of the former under the so-called scientific.

I do not intend to carry you through a long history of this disease, giving the minute details of conditions incident to cancer, but simply to confine myself to the description of carcinoma of the throat, and the history of the case under treatment.

I will give the definition of cancer of the throat as near as possible, so that we have in a nutshell the character of the disease which we have to treat, namely: A cancerous growth in the wall of the gullet, generally undergoing ulceration, but giving rise at the same time to great narrowing of the canal; often to perforation of the trachea or bronchi, and in rare instances to perforation of one of the large blood-vessels. In nearly all cases extreme dysphagia and marasmus are present.

Though cancer of the throat may be regarded as the typical disease of that organ, the affection with which most practitioners are best acquainted, it is not relatively common. According to Zenker and Ziemssen, in five thousand and seventy-nine autopsies, primary cancer of the gullet was present only thirteen times. Concerning the relative liability to cancer of the œsophagus as compared with other organs, there is less positive evidence. Dr. Walshe states that thirteen out of eight thousand two hundred and eighty-nine deaths from malignant disease in Paris were ascribed to cancer of the œsophagus.

The same causes which predispose to or excite cancer in other parts of the body lead to its development in the œsophagus. Among the former are heredity, age, and sex; among the latter, continued local irritation, accidental injury, and chronic inflammation may probably be reckoned. Heredity appears to have considerable influence, for among sixty cases examined by Dr. Morrell Mackenzie with reference to this circumstance, some member of the patient's family had died from malignant disease in eleven instances, while in ten cases observed by Richardson, there was in no instance wanting a history of some malignant affection among the relatives.

Age greatly influences the outlook of the disease, which is extremely rare under forty. The greatest number of cases are met with between fifty and sixty, although the decennia immediately before and after that period furnish almost as many cases.

The tubercular diathesis, which is ordinarily regarded as antagonistic to cancer in general, has been thought, on the contrary, by Lebert, Hamberzer, and Fritsche to predispose to that disease in the throat. Lebert observed the co-existence of pulmonary tubercle with cancer of the œsophagus in seven out of nine cases, while Béhier insists on the frequent coincidence of the two affections.

Among local causes, the abuse of spirits has, since the time of Gyser, been looked upon as an important factor in the production of œsophageal cancer, and the greater prevalence of the disease among men as compared with women has been attributed to this cause.

It is quite possible that the abuse of spirits may predispose in several ways to the development of cancer in the throat. Thus, by lowering the tone of the nervous system and causing degeneration of tissue, it may render all the organs less capable of resisting the constitutional taint. There does not, however, exist any decisive evidence on this point. Again, alcohol may directly irritate the mucous membrane, or indirectly produce a similar result by causing eructations and vomiting.

Further, when people are half intoxicated, they are apt to be careless as to what and how they eat, and under these circumstances pieces of meat or foreign bodies accidentally introduced into the food are more likely to be swallowed, and thus set up irritation. It is my opinion, that the effect of excessive indulgence in alcohol has been over-rated in considering the etiology of œsophageal cancer.

Symptoms: The most constant, striking, and important phenomenon is difficulty in swallowing. It is this which usually first attracts and then rivets the sufferer's attention. The train of symptoms is generally somewhat as follows: The patient first experiences an occasional obstruction to the descent of food, if he takes a large mouthful, or if the food is of a dry nature. In a short time this difficulty becomes habitual, and the patient complains that the food lodges somewhere, usually at the same point, when he tries to swallow. He now often begins to be troubled with cough, especially when deglutition is attempted, and as the disease progresses he is obliged to wash down every mouthful with a draught of liquid, and he soon finds that he cannot take solids at all, except after prolonged mastication and with the aid of fluids. Then he is no longer able to swallow solid food in any form, his diet is restricted to liquids, and he loses flesh rapidly.

As time goes on, in some cases, the stricture becomes so narrow that even liquids cannot be got down, or a fistulous opening being formed between the œsophagus and trachea, the swallowed liquids pass into the windpipe and are immediately ejected by a violent and painful attack of coughing. As soon as the gullet becomes much contracted the patient begins to spit up a frothy fluid, which is at first clear, and closely resembles saliva, but which soon becomes viscid and muco-purulent, and is not unfrequently streaked with blood.

Sometimes small particles are voided, which, on microscopic examination, are found to be of a cancerous nature. Emaciation rapidly advances, and the patient soon becomes greatly wasted, and so weak that he is unable to take any exercise, or induced to perform any act requiring muscular effort. The cancerous cachexia is often absent, the patient dying of starvation before the constitution becomes markedly perverted.

I will now return to the history of the case at hand. Mr. R. R., aged sixty years, consulted me February 12th, 1888, and narrated to me the following symptoms: Great difficulty in swallowing, especially when any solids were attempted; marked aphonia was present, although at times the voice had a piping tone. The general appearance of the patient at once suggested cancer. Associated with these few throat symptoms, none of which were very peculiar, he complained of sharp, cutting pains which constantly shifted from one part of the body to another. He was greatly emaciated, and almost too weak to walk. I made a careful examination of the throat with suitable throat mirrors, and from the appearance of the growth and the general symptoms present pronounced it cancer, not to the patient but to the son, who accompanied him.

The family and friends not being satisfied with my diagnosis, persuaded him to go see Professor D. Hayes Agnew, of Philadelphia, and in a letter which the doctor wrote me he confirmed the diagnosis.

My first prescription was Pulsatilla$^{52m}$, a few doses, followed by Sac. lac. for one week. No improvement now showing itself, and the patient complaining of being worse, Lac caninum was given for two weeks, with the same report. I now made a very careful examination of his symptoms: he complained, among other symptoms with those before mentioned, of sleeplessness and great aggravation if he happened to fall asleep and then awakened; with this marked sensitiveness of throat to contact was present. At this date, March 5th, Lachesis$^{cm}$ was given, a few doses for a week. The patient not improving, I determined to repeat the Lachesis and give it in a lower potency, which I did in the thirtieth dilution; this I repeated every three hours for two weeks, and marked improvement was manifest. Sac. lac. was again given for a number of days, and the improvement continued. I wrote to my friend, Dr. Tyrrell, of Toronto, to send me provings of Kali cyanate, if he had such, and if it were homœopathically indicated I intended to give it if Lachesis failed. He kindly sent me grafts of Lachesis in the higher dilutions and wrote me that the provings of Kali cyanate were not as yet collected. I gave my patient one dose of Lachesis$^{8mm}$—one of the potencies which he sent me—and he continued to improve until five weeks ago, when dysphagia set in and lasted for three days. During this time the patient was unable to swallow the least liquid or solid, not even the saliva, and was, as he says, nearly starving. Belladonna$^{cm}$ was now given, which relieved the spasmodic constriction of the œsophagus, after Hydrophobinum and Kali carb. had failed. He was now able to swallow, to use his own words, "better than for four months." After this marked improvement, which all the time, you must remember, continued while taking Lachesis, his strength had returned, color of face changed, and in every way improved, as he says, four hundred per cent. Sac. lac. was given, and improvement continued. About three weeks ago, the following symptoms were manifest: Throat felt as if filled up, as if he could not swallow; white, frothy, salty mucus raised when coughing; worse toward evening. Constipation; stools large, in hard lumps; when partly expelled it slips back again. I now concluded to give Silicea$^{cm}$, a few doses, which I did with continued improvement to this date. All the above symptoms have disappeared, and I contend that if cancerous conditions are curable they are only by strictly adhering to the law of *Similia, Similibus, Curantur.**
—*Clinical Bureau I. H. A.*

* A recent note from Dr. Allen informs us that this patient died about two months after the above report was made; the same alleviation continued up to the time of his death.—EDITORS.

